# Identification of potential biomarkers and therapeutic targets for osteoarthritis associated with arginine and proline metabolism based on transcriptome sequencing and bioinformatics

**DOI:** 10.3389/fgene.2026.1795267

**Published:** 2026-07-03

**Authors:** Xiao-Hua Chen, Jun Liu, Ling Qiu, Yang Zhan, Zhuo-Ming Zheng, Peng Chen, You-Xin Su, Jie-Mei Guo, Sheng-Jian Weng

**Affiliations:** 1 First Clinical Medicine College, Fujian University of Traditional Chinese Medicine, Fuzhou, China; 2 School of Orthopedics and Traumatology, Fujian University of Traditional Chinese Medicine, Fuzhou, China; 3 Department of Orthopedics, The Second General Hospital of Fuzhou, Fuzhou, China; 4 Key Laboratory of Orthopedics & Traumatology of Traditional Chinese Medicine and Rehabilitation, Ministry of Education, Fujian University of Traditional Chinese Medicine, Fuzhou, China

**Keywords:** arginine and proline metabolism, bioinformatics, osteoarthritis, synovial tissue, transcriptome sequencing

## Abstract

**Background and Objectives:**

Osteoarthritis (OA) is a chronic degenerative joint disease. Approximately 300 million people worldwide suffer from OA, which shows a high incidence in middle-aged and elderly populations, with a prevalence of 50% among individuals aged over 60 years. Its core clinical symptoms consist of joint pain, swelling, and dysfunction. Studies have shown that arginine and proline metabolism play an important role in the pathogenesis and progression of OA, but the specific mechanism is still unclear. This study aimed to identify biomarkers and drug therapeutic targets for OA associated with arginine and proline metabolism.

**Methods:**

Synovial tissues of healthy individuals and OA patients were collected for transcriptome sequencing, and the differentially expressed genes (DEGs) between the two groups were compared and analyzed. Arginine and proline metabolism-related genes (APRGs) were obtained from the molecular signature database. The candidate genes were identified by weighted gene co-expression network analysis (WGCNA), and then gene ontology (GO), Kyoto encyclopedia of genes and genomes (KEGG) pathway analysis and protein-protein interaction (PPI) were performed. Expression validation was performed using machine learning and ROC analysis to identify key genes. Gene set enrichment analysis (GSEA), immune cell infiltration, and drug prediction were used to explore the mechanism of key genes in OA and potential therapeutic drugs. Finally, clinical samples were experimentally validated through RT-qPCR experiments.

**Results:**

Two hub genes (MYOM2 and TCAP) involved in arginine and proline metabolism were identified. A nomogram constructed based on these genes indicated that MYOM2 and TCAP are key and reliable predictors for osteoarthritis risk. The RT-qPCR experiments on clinical samples showed that the expression levels of these hub genes were significantly downregulated in the synovial tissue of OA patients (p < 0.05), suggesting their potential as diagnostic biomarkers.

**Discussion:**

MYOM2 and TCAP are hub genes in OA metabolism with arginine and proline, which may become new diagnostic markers and potential therapeutic targets for OA.

## 1 Introduction

Osteoarthritis (OA) is a degenerative joint disease with joint pain and dysfunction as the main symptoms, and is one of the most common causes of chronic pain and disability in the elderly (Yuan et al., 2020; Molnar et al., 2021) which has attracted wide attention due to its high prevalence and high disability rate. According to statistics, about 300 million people around the world suffer from OA (Abramoff and Caldera, 2020), which is particularly common in middle-aged and elderly people. The prevalence rate can reach 50% in people over 60 years old, and can reach 80% in people over 75 years old, and the disability rate can reach 53% (Quicke et al., 2022), which not only impairs patients’ quality of life, but also brings heavy burden to families and society. It is understood that genetics, age, obesity, trauma, inflammation, and metabolic changes are all considered as risk factors for OA (Chaganti and Lane, 2011). However, so far, the etiology and pathogenesis of OA have not been fully elucidated, and current treatment strategies, including oral non-steroidal anti-inflammatory drugs (NSAIDs), intra-articular injection, and joint arthroplasty, mainly focus on pain alleviation. However, pharmacological interventions are associated with gastrointestinal, cardiovascular, and other adverse reactions, whereas surgical treatment carries a risk of postoperative infection (Bourget-Murray et al., 2023). Therefore, identifying diagnostic and therapeutic targets for OA continues to be a key focus of ongoing research. Our study aims to advance OA treatment by elucidating novel biomarkers and effective drug targets.

Amino acids (AAs) are small molecule metabolites, which not only participate in the construction of peptides and proteins, but also play an important role in immune function, cytokine secretion, and response. Some studies using ultra-high performance liquid chromatography-quadrupole time-of-flight mass spectrometry found that there were differences in AAs spectra between healthy controls and OA patients, and arginine and proline metabolism were the most significantly affected metabolic pathways (Chen et al., 2018). A recent multi-omics analysis of synovial tissue and synovial fluid has demonstrated localized dysregulation of arginine metabolism in the osteoarthritic joint, as evidenced by significantly elevated arginine levels in the synovial membrane and enrichment of the arginine biosynthesis pathway in the synovial fluid, a pattern of local accumulation and enhanced biosynthetic activity that promotes cartilage degradation and synovial inflammation (Ge et al., 2025; Wang et al., 2026). Proline, the other key amino acid in this pathway, is essential for collagen synthesis and the maintenance of extracellular matrix stability (Karna et al., 2020). A recent study has demonstrated that the circular RNA circUbqln1 may impair proline metabolism in OA by modulating PRODH activity, thereby inhibiting collagen synthesis and compromising the structural integrity of articular cartilage (Feng et al., 2024). These findings indicate that arginine accumulation and pathway enrichment in synovial tissues promote cartilage degradation and synovial inflammation, while proline dysregulation inhibits collagen synthesis and compromises cartilage structural integrity. It has been demonstrated that arginine and proline metabolism play an important role in the occurrence and development of OA. Therefore, the identification of key biomarkers and potential therapeutic targets related to arginine and proline metabolism can provide positive value for the diagnosis and treatment of OA.

**FIGURE 1:**
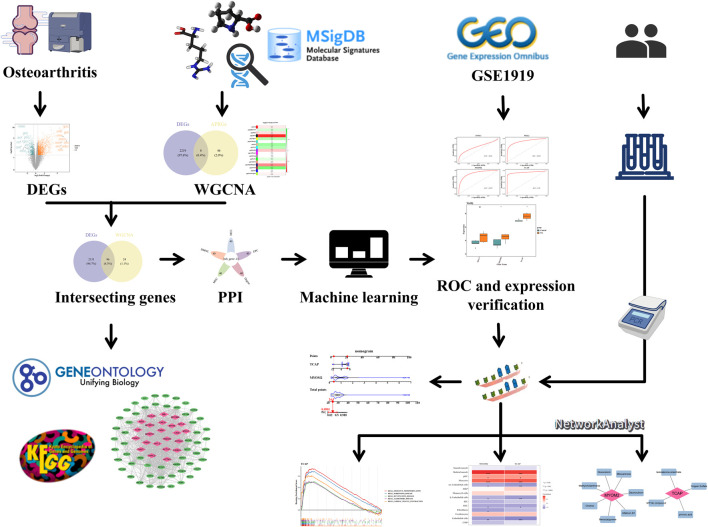
The flowchart of this study. Synovial tissue samples were collected from OA patients and healthy controls at the Second General Hospital of Fuzhou (training set: 12 OA patients and 10 controls) for transcriptome sequencing. An external GEO dataset (GSE 1919; 5 OA, 5 controls) served as validation, and 54 APRGs were obtained from MSigDB. DEGs were identified using DESeq2 (|log2FC| > 0.5, p < 0.05), intersected with APRGs, and scored by ssGSEA. WGCNA on APRG ssGSEA scores identified key modules, from which candidate genes were derived and analyzed via GO/KEGG enrichment and STRING/Cytoscape PPI hub screening. Random forest and ROC analyses selected key diagnostic genes, further assessed by GSEA, xCell-based immune infiltration, and a nomogram model. Hub genes were experimentally validated by RT-qPCR, and potential compounds were predicted (DSigDB/Enrichr) and evaluated via molecular docking (CB-Dock/AutoDock Vina) and 100-ns GROMACS MD simulations.

Fortunately, in recent years, the development of transcriptome sequencing technology and the application of bioinformatics in the medical field have provided new approaches for studying the molecular mechanisms of various diseases. We collected synovial tissue data from healthy individuals and OA patients. Through the differential expression analysis of transcriptome sequencing and weighted gene co-expression network analysis (WGCNA), candidate genes associated with arginine and proline metabolism were identified and functionally characterized. Multiple bioinformatics methods were further employed to screen and pinpoint hub genes, enabling efficient determination of the functions and roles of differentially expressed genes. These findings were subsequently validated experimentally. Additionally, the pharmacological activities of two potential OA drug targets were verified via drug prediction, molecular docking, and molecular dynamics simulations, offering novel directions and potential therapeutic targets for the prevention and treatment of OA (Figure 1).

## 2 Materials and methods

### 2.1 Synovial tissue sample collection and sequencing

Human synovial tissue samples were collected from OA patients and healthy controls from the Fuzhou Second General Hospital. The training set included 12 OA patient samples and 10 normal control samples, all from human donors. This research was approved by the Hospital Ethics Committee (Ethics Number: 2021014). All patients have signed the informed consent form. All operations were carried out strictly in accordance with the relevant guidelines and regulations of the hospital.

As per the manufacturer’s protocol, TRIzol reagent (Invitrogen, CA, USA) was utilized to separate and purify the total RNA from synovial tissue samples. RNA quantity and purity were evaluated with a NanoDrop ND-1000 spectrophotometer (NanoDrop, Wilmington, DE, USA), and its integrity was tested via a Bioanalyzer 2,100 system (Agilent, CA, USA). Validation was further confirmed by agarose gel electrophoresis. For downstream analyses, samples were required to have a concentration over 50 ng/μL, RIN values above 7.0, OD260/280 ratios higher than 1.8, and total RNA amounts greater than 1 μg. After two rounds of purification with oligo (dT) magnetic beads (Dynabeads Oligo (dT), Thermo Fisher, USA), polyadenylated mRNA was isolated. The mRNA fragmentation was achieved at 94 °C for 5–7 min via the NEBNext® Magnesium RNA Fragmentation Module. The fragmented RNA was reverse transcribed into cDNA with the help of SuperScript™ II Reverse Transcriptase (Invitrogen). Double-stranded cDNA was synthesized via *E. coli* DNA polymerase I and RNase H (NEB), incorporating dUTP Solution (Thermo Fisher) to generate blunt-ended fragments. A single adenine base was added to the 3′ends to facilitate ligation with adapters containing a complementary thymine base. The library was purified and size-selected with the help of magnetic beads. Subsequently, double-stranded DNA was treated via UDG enzyme (NEB) to remove uracil residues. The DNA library underwent PCR amplification under the following conditions: 3-min denaturation at 95 °C, 8 cycles of 98 °C for 15 s, 60 °C for 15 s, and 72 °C for 30 s, finishing with a 5-min extension at 72 °C. A library with an average fragment size of 300 ± 50 bp was generated. Paired-end sequencing was subsequently performed on the Illumina Novaseq™ 6,000 platform (LC Bio Technology CO., Ltd., Hangzhou., China) with a 150 bp read length mode according to the manufacturer’s standard protocol. Raw sequencing reads were generated and archived in FASTQ format. Quality of the data was assessed with FastQC (v. 0.11.9) to remove low-quality reads (Brown et al., 2017). Phred quality scores were used to evaluate base call accuracy, with a score ≥30 defined as acceptable, and nucleotide distribution was analyzed to detect any AT/CG bias. Clean reads were subsequently mapped to the human reference genome, which was downloaded via the Ensembl database (https://asia.ensembl.org/info/about/species.html), using Hierarchical Indexing for Spliced Alignment of Transcripts 2 (Hisat2). Transcripts assembly were carried out with Cufflinks (v. 2.2.1) to produce count data (Trapnell et al., 2012). To assess sample clustering and distinguish between OA and control groups, principal component analysis (PCA) was executed with the help of “scatterplot3d” (v. 0.3–42, Li et al., 2022).

### 2.2 Gene expression dataset screening and processing

For the testing set, the GSE 1919 (GPL91) dataset via the GEO database (https://www.ncbi.nlm.nih.gov/geo/) was used, which encompassed 5 synovial tissue normal control samples and 5 OA patient samples. Additionally, 54 APRGs were extracted via the Molecular Signatures Database (MSigDB) (https://www.gsea-msigdb.org/gsea/msigdb/), specifically from the KEGG arginine and proline metabolism gene set (Wang et al., 2023). Gene expression data were subjected to Median Ratio Normalization using the DESeq2 (v. 3.50.3, Pang et al., 1989) package to ensure the comparability of gene expression levels among samples. Once the data were quality-controlled, raw counts were normalized for library size using the DESeq2 estimate size factors function (median-of-ratios method), and the normalized counts were used for downstream analysis.

### 2.3 Differential expression analysis

The DESeq2 package was employed to identify DEGs by comparing OA and control samples within the training set [|log2fold change (FC)| > 0.5 and p < 0.05]. Based on log2FC, most 10 upregulated and downregulated genes were established and ranked in descending order, subsequently highlighted in the volcano plot via the “ggplot2” R package (v. 3.3.6, Gustavsson et al., 2022), and a heatmap was built by the “ComplexHeatmap” R package (v. 2.14.0, Gu and Hübschmann, 2022).

### 2.4 Weighted gene Co-expression network analysis (WGCNA)

After identifying DEGs, the intersection of APRGs and DEGs was taken to pinpoint the differentially expressed APRGs in OA versus control groups. Using all samples within the training set, single-sample Gene Set Enrichment Analysis (ssGSEA) scores for the differentially expressed APRGs were processed via “GSVA” (v. 1.42.0, Hänzelmann et al., 2013). A Wilcoxon test was adopted to compare differences in APRG scores across the OA and control cohorts (p < 0.05). Subsequently, WGCNA was performed on the APRGs scores using the “WGCNA” R package (v. 1.71, Langfelder and Horvath, 2008) for building a co-expression network and detecting modules within the training set. First, hierarchical clustering was performed on the training set using the hclust function to identify potential deviant samples. Outliers were omitted to safeguard the accuracy of upcoming analyses. After outlier removal, the ssGSEA scores of APRGs for each sample were introduced, and hierarchical clustering was re-executed via the hclust function. To construct a co-expression network, the pickSoftThreshold function was harnessed to determine optimal soft-threshold power, ensuring that the network maximally approximated a scale-free topology. A soft-threshold parameter was selected from a range of 1 to 30, with the goal of identifying the appropriate threshold for gene-gene correlations. The *R* ^2^ threshold was set to 0.85, ensuring a scale-free network with lower mean connectivity. To better represent gene interaction strength and similarity, a soft-threshold power was chosen to maintain a scale-free topology, refining the accuracy of the adjacency matrix and topological overlap matrix (TOM). Based on the TOM, a gene dendrogram was generated using hierarchical clustering to group genes into modules. The dynamic tree-cutting algorithm identified modules, specifying a minimum size of 100 genes. Finally, modules were merged based on a height cutoff of 0.4, resulting in candidate modules for further analysis.

### 2.5 Identification of candidate genes

Further correlation analysis was performed between the ssGSEA scores of APRGs and the gene modules. Key modules with the strongest positive and negative correlations (|cor| > 0.3, p < 0.05) were opted for deeper exploration. Subsequently, module membership (MM) and gene significance (GS) were calculated for key module genes. MM represents the correlation between genes and their respective modules, while GS reflects the correlation between genes and the trait of interest. Scatter plots were rendered via “ggplot2”. Key module genes were those with |MM| above 0.9 and |GS| above 0.6. A Venn diagram was generated via “ggVenn” (v. 1.2.2, Zheng et al., 2022) to identify the intersection between DEGs and APRGs-related key module genes, overlapping genes were pinpointed as candidate genes.

### 2.6 Functional analysis of candidate genes

Then, candidate genes underwent gene ontology (GO) and Kyoto encyclopedia of genes and genomes (KEGG) pathway analysis (p < 0.05) via the “clusterProfiler” R package (v. 4.7.1.003, Wu et al., 2021), with the human gene set from the “org.Hs.e.g.,.db” as the background. GO analysis was performed across 3 categories: biological process (BP), cellular component (CC), and molecular function (MF), and 5 most significant genes from each section enriched pathways were rendered via “GOplot” (v. 1.0.2, Walter et al., 2015). Similarly, the KEGG pathway analysis results were also visualized with the “GOplot” R package. To explore PPI, the candidate genes were uploaded to the search tool for the retrieval of interacting the genes (STRING) database (https://cn.string-db.org/), the PPI network was established with a confidence score threshold of 0.40. Finally, PPI networks were visualized via Cytoscape software (v. 3.9.1, Shannon et al., 2003). The top 40 candidate genes were selected from five algorithms individually: Degree, Density of Maximum Neighborhood Component (DMNC), Closeness, Clustering Coefficient, as well as Edge Percolated Component (EPC). The intersecting genes from these selections were used for subsequent analysis.

### 2.7 Screening for key biomarkers

The intersecting genes were subjected to machine learning using random forest analysis by “randomForest” (v. 4.7–1.1, Alderden et al., 2018) to identify feature genes. The top 5 feature genes, ranked by their importance based on the mean decrease in the Gini index, were selected for further experiments. To assess their ability to differentiate OA samples from control samples, receiver operating characteristic (ROC) curves were implemented through “pROC” (v. 1.18.5, Robin et al., 2011), and area under the curve (AUC) was quantified on both databases. Feature genes with AUC values greater than 0.7 were evaluated for expression differences between OA and control in both databases using the Wilcoxon test (p < 0.05). Box plots were generated using “ggplot2” to visualize differential expression (p < 0.05). Genes with marked differential expression between groups and consistent trends across both datasets were identified as hub genes.

### 2.8 Creation and evaluation of nomograms based on hub genes

In the training set, a nomogram model was built from the expression levels of hub genes, with OA samples considered as the outcome event. The “rms” R package (v. 6.3–0, Sachs, 2017) was used to construct this nomogram. To evaluate the model’s predictive ability, calibration curves were generated via “rms”. A p-value >0.05 and a slope close to 1 indicated higher predictive accuracy. Additionally, ROC curves rendered through “pROC”, with an AUC >0.7 considered indicative of good model accuracy.

### 2.9 Gene set enrichment analysis (GSEA)

GSEA was executed with the help of gene sets from the MSigDB database, with the KEGG background gene set from “org.Hs.e.g.,.db”. For each of the hub genes in the training set, correlations were calculated across hub genes and all remaining genes to generate a prioritized catalog of associated genes. Then, GSEA was conducted based on this ranking, with the significance threshold set at p. adjust <0.05 as well as |Normalized Enrichment Score (NES)| > 1.

### 2.10 Immune infiltration assessment

In the training set, enrichment scores of 64 immune cell types were employed to highlight variation between OA and control samples via the xCell algorithm implemented through the “xCell” R package (v. 1.1.0, Aran et al., 2017). Next, the Wilcoxon test (p < 0.05) was used to identify significantly different immune cell types between the OA and control groups. To gain more insight into the interaction between hub genes and significantly different immune cells, Spearman correlation analysis was done on all samples in the training set via “psych” (v. 2.2.9, Robles-Jimenez et al., 2021). |cor| > 0.3 and p < 0.05 were regarded as evidence of a correlation, and a moderate correlation (|cor| > 0.38) was considered statistically significant.

### 2.11 Drug prediction, molecular docking, and molecular dynamics simulation

To evaluate the clinical potential of hub genes, the Drug Signatures Database (DSigDB) (http://tanlab.ucdenver.edu/DSigDB, accessed on 10 November 2025) within EnrichR (v. 3.2, Sheng et al., 2025) was utilized in this study to predict candidate compounds interacting with hub genes. The compound with the highest combined score for each key gene was selected as the ligand for molecular docking analysis. The 3D structures of hub genes and their corresponding compounds were retrieved from the RCSB PDB database (https://www.rcsb.org/) and PubChem database (https://pubchem.ncbi.nlm.nih.gov/), respectively. Molecular docking was performed using the CB-Dock online server (https://cadd.labshare.cn/cb-dock/php/index.php), a tool implementing blind docking based on AutoDock Vina that can automatically identify binding pockets and calculate binding free energy. To verify the stability of the docked conformations, human-derived protein sequences were further obtained from NCBI, and 100 ns molecular dynamics (MD) simulations were conducted using GROMACS 2024.2 software (the simulated systems included MYOM2-MELPHALAN and TCAP-Testosterone Enanthate). In the MD simulations, the AMBER14SB force field and GAFF force field were employed to generate parameters and topology files for proteins and small-molecule ligands, respectively. The system was placed under periodic boundary conditions, and the simulation box was solvated with TIP3P water molecules. To maintain system electroneutrality, 0.15 mol/L Na^+^ and Cl^−^ ions were added. Energy minimization was accomplished using the steepest descent method. Subsequently, two-stage pre-equilibration was performed: first, 100 ps temperature equilibration at 300 K under the NVT ensemble, followed by 100 ps pressure equilibration at 1 bar under the NPT ensemble. MD simulations were performed to evaluate the root mean square deviation (RMSD), root mean square fluctuation (RMSF), total energy, and number of hydrogen bonds of the complexes, respectively.

### 2.12 RT-qPCR analysis of synovial tissue in human OA

The Real-time quantitative polymerase chain reaction (RT-qPCR) experiment set included OA patient samples and normal control samples, all self-collected from human donors. Subsequently, the synovial tissues collected from clinical samples were minced with bone forceps and then pulverized into fine powder using a mortar pre-cooled with liquid nitrogen. Total RNA was extracted from each tissue sample following the protocol of the TRIzol kit, and the RNA concentration was quantified. Using the reverse transcriptase HiScript Q RT SuperMix for qPCR (+gDNA wiper), the reverse transcription system was prepared as per the manufacturer’s instructions, residual genomic DNA was removed, and complementary DNA (cDNA) was synthesized via reverse transcription. Subsequently, 2 mL cDNA was incorporated into the ChamQ SYBR qPCR Master Mix reaction system for RT-qPCR. The thermal cycling conditions comprised denaturation at 95 °C for 30 s, annealing at 95 °C for 10 s, and extension at 60 °C for 30 s for a total of 40 cycles. Following the acquisition of CT values for MYOM2 and TCAP genes in each group, the 2^−ΔΔCT^ method was used to determine the relative expression levels of each gene. Primer sequences are detailed in Table 1.

**TABLE 1:** The gene primer sequences of MYOM2 and TCAP.

Gene	Primer sequence (5'→3′)		Length (bp)
MYOM2	Forward primer Reverse primer	ATGGCGTACACACACTGGAG ACCGAACGGAATGGTTCCTC	151
TCAP	Forward primer Reverse primer	CATGAGACCTACCACCAGCA ATCTTGGCAGGGGTGAAGAT	155
GAPDH	Forward primer Reverse primer	GGTGTGAACCATGAGAAGTATGA GAGTCCTTCCACGATACCAAAG	123

## 3 Results

### 3.1 APRG-associated key modules reveal dysregulated genes in OA

**FIGURE 2:**
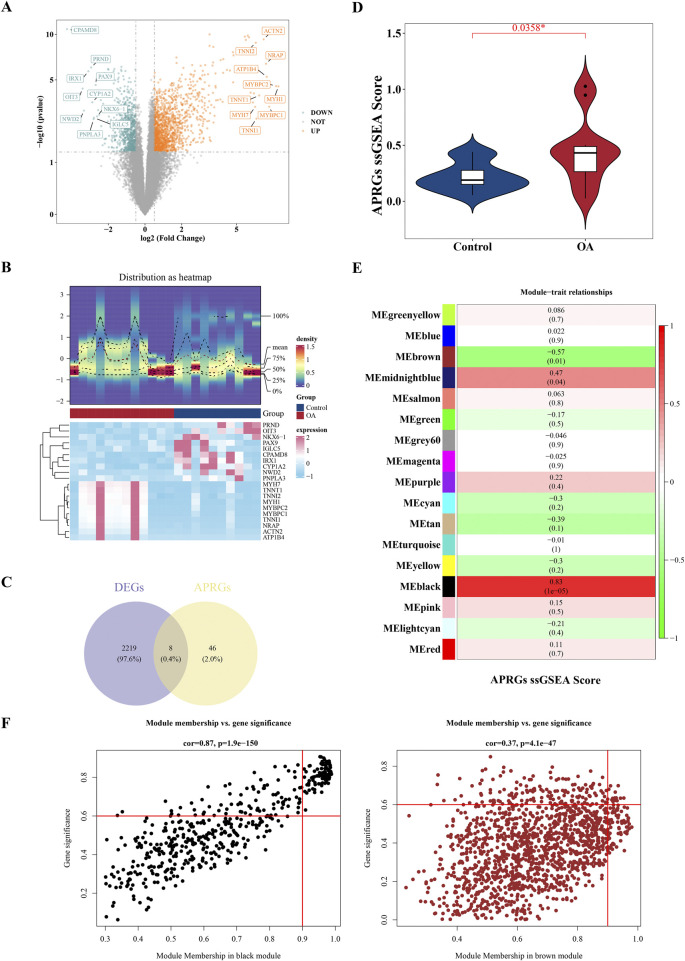
Identification of key module genes related to arginine and proline metabolism in OA. **(A,B)** Volcano map and differential gene heatmap. In total, 2,227 differentially DEGs were obtained by comparing OA with control samples, consisting of 1,435 upregulated and 792 downregulated genes in OA. **(C)** Intersection of APRGs and differential genes. 54 APRGs were acquired from the MSigDB, and intersection with the 2,227 DEGs screened out 8 differentially expressed APRGs in OA versus control groups. **(D)** Analysis of differences in APRGs scores between OA group and control group. The ssGSEA scores of APRGs were markedly higher in OA than in controls (p < 0.05). After removing outliers, APRG ssGSEA scores were recalculated for each sample, followed by re-performing clustering analysis. **(E)** Heat map of correlation between APRGs ssGSEA score and key modules. MEblack and MEbrown modules were identified as key modules. MEblack exhibited the strongest positive correlation with APRG scores (r = 0.83, p < 0.001), while MEbrown showed the most prominent negative correlation (r = −0.57, p < 0.05). **(F)** Scatter plot of correlation between module members and module/module traits. The MEblack and MEbrown modules presented strong module–trait correlations, with r = 0.87 and r = 0.37, respectively.

After quality control, no AT/CG separation was observed (Supplementary Figure S1A). The transcriptome data from self-collected synovial tissue samples showed a mapping rate above 90% for all samples, confirming high data quality (Table 2). PCA revealed clear separation between OA and control samples, indicating distinct transcriptomic profiles in two groups (Supplementary Figure S1B). Differential expression analysis comparing OA and control samples revealed 2,227 DEGs. Specifically, 1,435 genes with increased expression and 792 with decreased expression in OA were identified (Figures 2A,B). The 54 APRGs were obtained from the MSigDB. By intersecting 54 APRGs with the 2,227 DEGs, 8 differentially expressed APRGs were identified comparing OA and control groups (Figure 2C). The ssGSEA scores of APRGs were markedly elevated in the OA group relative to the control group (p < 0.05), suggesting a strong correlation between APRG expression and OA (Figure 2D). Outlier samples in this study were excluded. Following sample exclusion, ssGSEA scores of APRG were recalculated for each sample, and clustering analysis was performed (Supplementary Figure S1C,D). A soft threshold of R2 = 0.85 was established, and a power value of 24 was chosen to achieve a scale-free network, with a low mean connectivity. Thus, the optimal soft threshold (β) was set to 24 (Supplementary Figure S1E). Correlation analysis identified 17 modules associated with the ssGSEA scores of APRGs (Supplementary Figure S1F). Among these, the MEblack and MEbrown modules were identified as key modules, with MEblack showing the top positive correlation (cor = 0.83, p < 0.001) and MEbrown showing the largest negative correlation (cor = −0.57, p < 0.05) with APRG scores (Figure 2E). A total of 120 key module genes for further analysis, with 105 black module and 15 brown module genes, with MEblack and MEbrown modules showing strong module membership–trait correlations (MEblack: cor = 0.87; MEbrown: cor = 0.37) (Figure 2F).

**TABLE 2:** Comparison rate of each sample.

Sample	Total_reads	Mapped (%)
OA2	35,827,848	90.31%
OA3	36,794,650	93.55%
OA4	36,761,617	92.76%
OA5	33,206,496	94.10%
OA6	37,609,448	93.93%
OA7	37,404,510	92.16%
OA8	36,850,128	93.79%
OA9	38,197,749	94.08%
OA10	38,467,259	93.31%
OA11	35,937,128	90.11%
OA12	34,028,720	91.72%
OA13	40,332,314	93.48%
control1	36,567,931	93.80%
control2	32,318,946	93.52%
control3	37,786,353	94.26%
control4	36,040,191	94.58%
control5	31,607,790	93.77%
control6	31,087,738	93.23%
control7	38,281,588	94.84%
control8	38,171,818	94.14%
control10	34,778,731	93.58%
control11	48,291,671	93.17%

### 3.2 Candidate genes converge on muscle-related pathways

**FIGURE 3:**
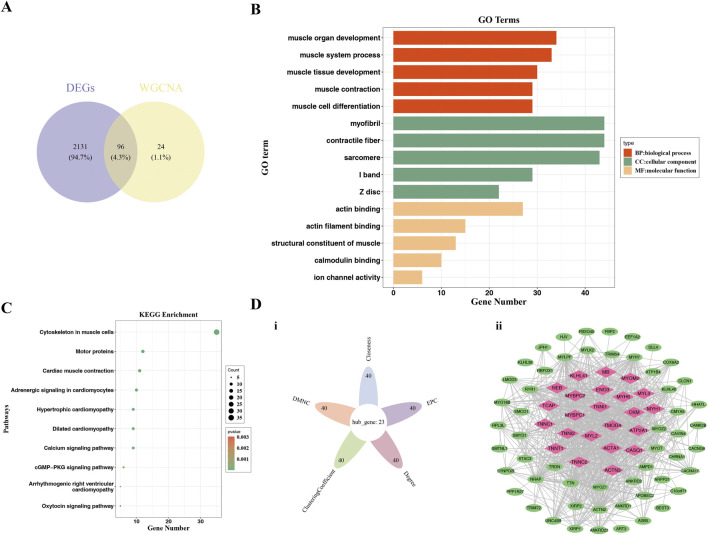
Identification of 96 candidate genes and their key pathways. **(A)** Venn diagram showing the intersection of 2,227 DEGs and 120 key module genes, yielding 96 candidate genes for downstream analysis. **(B)** GO enrichment analysis of candidate genes (p < 0.05); the top enriched terms are shown across three categories: biological process (BP; e.g., muscle organ development, muscle system process), cellular component (CC; e.g., contractile fiber, sarcomere), and molecular function (MF; e.g., actin binding). **(C)** KEGG pathway enrichment analysis identifying 15 significantly enriched pathways among candidate genes, including cytoskeleton in muscle cells, cardiac muscle contraction, and hypertrophic cardiomyopathy (p < 0.05). **(D)** Identification of hub genes and PPI network visualization. Venn diagram showing the overlap of the top 40 genes identified by five network topology algorithms (Degree, DMNC, Closeness, Clustering Coefficient, and EPC), yielding 23 hub genes (I). PPI network of candidate genes constructed using the STRING database (confidence score ≥0.40) and visualized in Cytoscape; nodes represent proteins and edges represent interactions. The 23 hub genes are highlighted in pink, while non-hub genes are shown in green (ii).

By intersecting the 2,227 DEGs, 120 key module genes, 96 genes were identified and designated as candidate genes (Figure 3A). GO enrichment analysis identified 302 terms, including 209 BP, 41 CC, 52 MF. BP terms were mainly enriched in muscle-related processes (muscle organ development, muscle system process, and muscle tissue development); CC terms in contractile structures (contractile fiber, myofibril, and sarcomere); and MF terms in actin binding and structural constituents of muscle (Figure 3B). The KEGG pathway enrichment analysis identified 15 significantly enriched pathways, including cytoskeleton in muscle cells, motor proteins, cardiac muscle contraction, adrenergic signaling in cardiomyocytes, and hypertrophic cardiomyopathy (Figure 3C). The PPI network was composed of 77 nodes and 890 edges. Using overlap of top 40 genes from each of the five algorithms, 23 intersecting genes were identified (Figure 3Di–ii).

### 3.3 MYOM2 and TCAP serve as hub diagnostic biomarkers in OA

**FIGURE 4:**
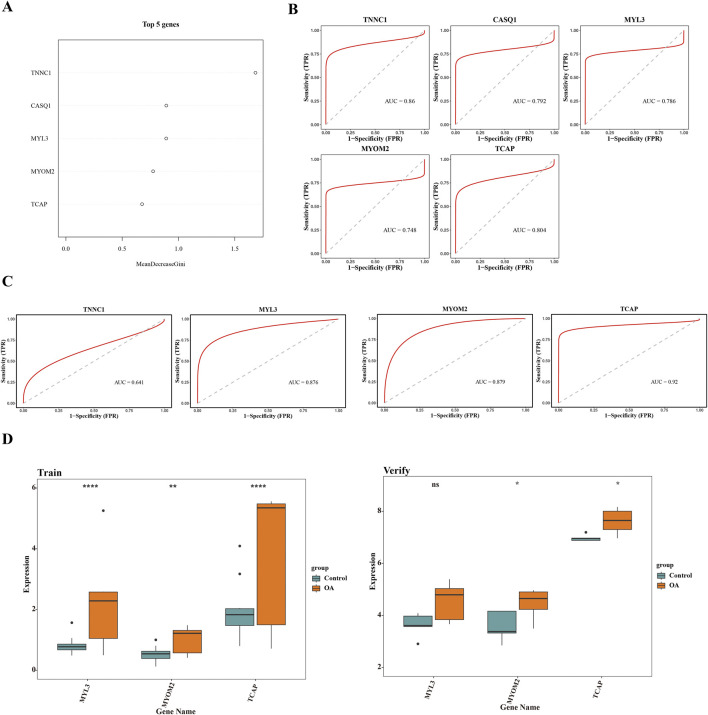
Screening of Diagnostic Biomarkers. **(A)** The top 5 feature genes (TNNC1, CASQ1, MYL3, MYOM2, TCAP) identified by random forest analysis, ranked by mean decrease in Gini index. The full variable importance plot is provided i Supplementary Figure S2. **(B,C)** Receiver operating characteristic (ROC) curves for the five feature genes in the training set **(B)** and testing set **(C)**; genes with AUC >0.7 in both datasets were retained. **(D)** Box plots showing the differential expression of MYOM2 and TCAP between OA and control groups in the training and testing sets; both genes were significantly downregulated in OA samples across both datasets (Wilcoxon test, p < 0.05), suggesting their role as hub genes.

A random forest analysis was performed on the 23 intersecting genes, and five feature genes (TNNC1, CASQ1, MYL3, MYOM2, and TCAP) were finally screened out (Figure 4A). The full variable importance plot is provided in Supplementary Figure S2. In the training set, all five feature genes had an AUC greater than 0.7 (Figure 4B). In the testing set, CASQ1 was not identified, TNNC1 had AUC values less than 0.7, and three feature genes (MYL3, MYOM2, and TCAP) had AUC values greater than 0.7 (Figure 4C). Therefore, these three feature genes (MYL3, MYOM2, and TCAP) were selected for expression validation. Of these 3 feature genes, MYL3 did not show significant expression in the testing set. However, MYOM2 and TCAP were significantly differentially expressed in both sets (p < 0.05), with consistent expression trends (Figure 4D). Therefore, MYOM2 and TCAP were confirmed as OA hub genes.

### 3.4 MYOM2 and TCAP nomogram showed promising predictive performance for OA risk

**FIGURE 5:**
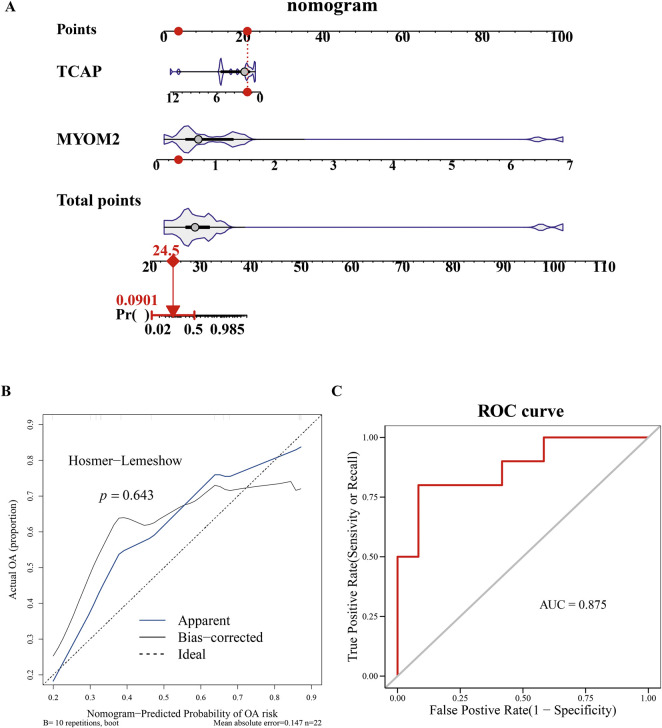
Risk prediction of hub genes in OA. **(A)** Nomogram for OA risk prediction. **(B)** Calibration curve was performed to verify model accuracy through intergroup comparison of uncorrected and bias-corrected data. No significant difference was observed (P = 0.643). **(C)** ROC curve analysis presented an AUC of 0.875, verifying robust fitting capability and high predictive efficacy of the nomogram.

Following the expression analysis of MYOM2 and TCAP, a nomogram was assembled to predict OA risk (Figure 5A). The calibration curve was used to further verify the accuracy of the model, with intergroup comparisons performed between uncorrected data and bias-corrected data. The results showed that p = 0.643 > 0.05, suggesting no statistically significant difference between the two groups. This finding confirms favorable result consistency before and after bias correction, and showed the satisfactory calibration capability and reliability of the nomogram (Figure 5B). Additionally, the ROC curve yielded an AUC of 0.875, showing a strong fit and high predictive performance for the nomogram (Figure 5C). These results suggested that MYOM2 and TCAP could be dependable hub genes for predicting OA risk.

### 3.5 MYOM2 and TCAP are enriched in neurodegenerative and cardiac pathways

**FIGURE 6:**
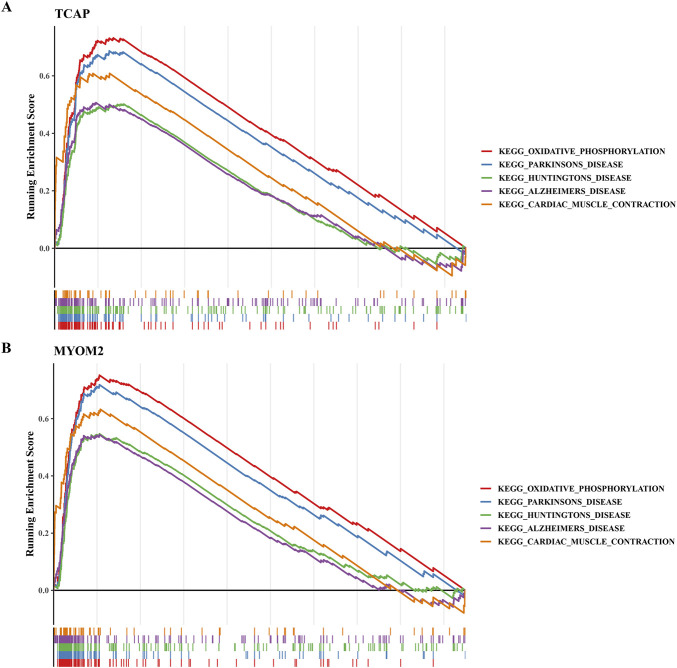
GSEA analysis of hub genes. **(A)** 5 TCAP-related pathways were significantly enriched, namely, Alzheimer’s disease, Parkinson’s disease, cardiac muscle contraction, oxidative phosphorylation, and Huntington’s disease. **(B)** 5 MYOM2-related pathways were significantly enriched, namely, Alzheimer’s disease, Parkinson’s disease, cardiac muscle contraction, oxidative phosphorylation, and Huntington’s disease.

GSEA revealed the functions of TCAP, showing its involvement in 5 signaling pathways (Figure 6A), while MYOM2 was enriched in 5 signaling pathways (Figure 6B). Both genes were commonly associated with pathways such as Alzheimer’s disease, Parkinson’s disease, cardiac muscle contraction, oxidative phosphorylation, and Huntington’s disease. The enrichment with MYOM2 and TCAP in these pathways suggested their potential role in OA, possibly by regulating cellular metabolism, oxidative stress response, and muscle function, thereby influencing the onset and progression of OA.

### 3.6 MYOM2 and TCAP correlate with skeletal muscle cells and endothelial cells in OA

**FIGURE 7:**
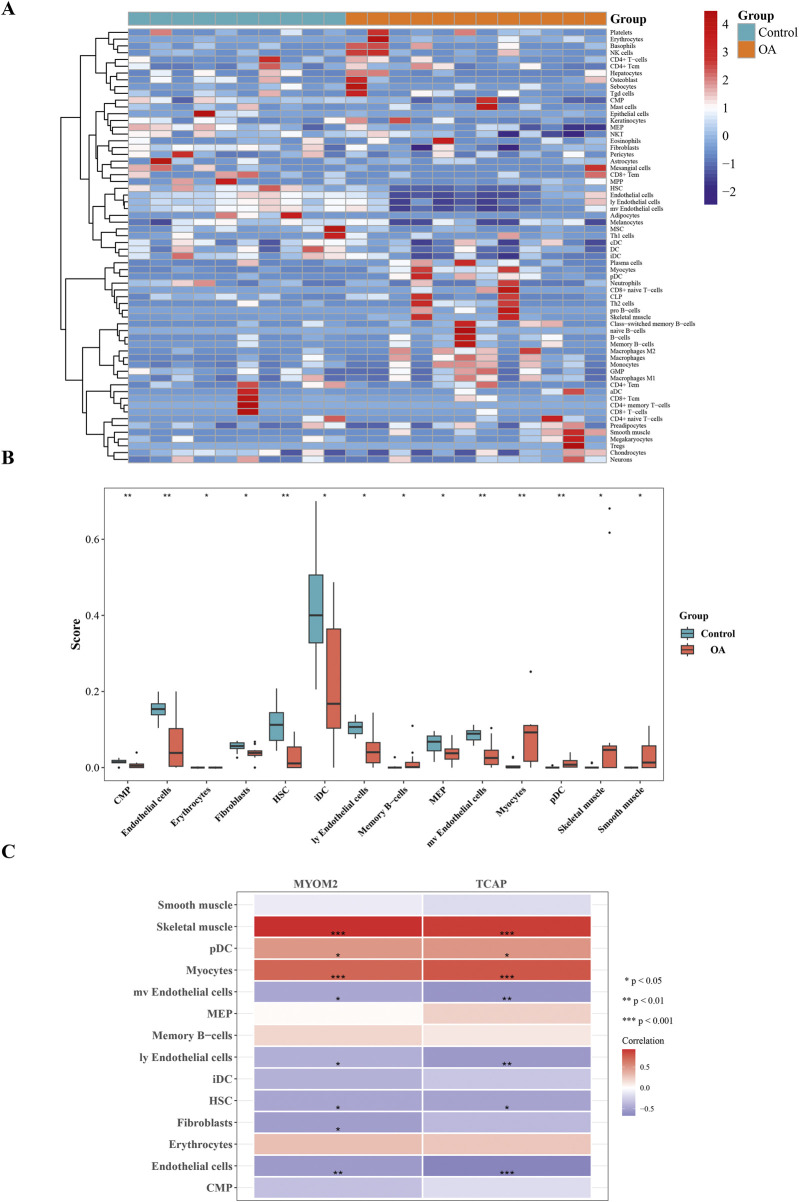
The correlation between immune cell infiltration and MYOM2, TCAP. **(A)** Heat map of expression of 64 immune cells in OA and control groups, with color coding normalized by row (z-score per cell type) to enable cross-sample comparison within each immune population. **(B)** Differential expression of 14 immune cells between OA and control groups. **(C)** Heat map of correlation between biomarkers and differential immune cells. Smooth muscle, skeletal muscle, pDC, memory B cells, and myocytes were more abundant in the osteoarthritis group.

A heatmap was generated to visualize the enrichment scores of 64 immune infiltrating cells in OA samples and normal controls (Figure 7A). Among these, 14 immune cells exhibited significant differences between the OA and control groups, and were defined as differential immune cells, such as common myeloid progenitors (CMP), endothelial cells, erythrocytes, fibroblasts, hematopoietic stem cells (HSC), immature dendritic cells (iDC), and lymphatic endothelial cells (Figure 7B). Notably, smooth muscle, skeletal muscle, pDC, memory B cells, and myocytes were more enriched in the OA group. Further correlation analysis revealed that TCAP had the strongest negative connection to endothelial cells (cor = −0.66, p < 0.001) and the strongest positive relationship with skeletal muscle cells (cor = 0.88, p < 0.001). Similarly, MYOM2 showed a substantial positive link to skeletal muscle cells (cor = 0.92, p < 0.001) and a notable negative connection with endothelial cells (cor = −0.54, p < 0.01) (Figure 7C; Table 3).

**TABLE 3:** Correlation results of immune cells.

Gene	Cell	Correlation	P value
MYOM2	CMP	−0.339,356,296	0.122327878
TCAP	CMP	−0.178994918	0.425438244
MYOM2	Endothelial cells	−0.543349358	0.008963843
TCAP	Endothelial cells	−0.664783986	0.000737658
MYOM2	Erythrocytes	0.305978423	0.166091029
TCAP	Erythrocytes	0.269235672	0.225648839
MYOM2	Fibroblasts	−0.523016118	0.012503075
TCAP	Fibroblasts	−0.37616494	0.084453917
MYOM2	HSC	−0.479616871	0.023899026
TCAP	HSC	−0.500568258	0.017656713
MYOM2	iDC	−0.410502541	0.057741205
TCAP	iDC	−0.304347826	0.16847502
MYOM2	Endothelial cells	−0.424738791	0.048797687
TCAP	Endothelial cells	−0.551821541	0.007756195
MYOM2	Memory B-cells	0.19566495	0.382849291
TCAP	Memory B-cells	0.114185704	0.612873626
MYOM2	MEP	0.026546174	0.90665028
TCAP	MEP	0.220276767	0.324599577
MYOM2	Mv endothelial cells	−0.477131564	0.024743739
TCAP	Mv endothelial cells	−0.575381141	0.005083271
MYOM2	Myocytes	0.716310912	0.000177055
TCAP	Myocytes	0.784827608	1.53E-05
MYOM2	pDC	0.490472102	0.020475709
TCAP	pDC	0.499026848	0.018065556
MYOM2	Skeletal muscle	0.923240189	9.31E-10
TCAP	Skeletal muscle	0.879631579	6.95E-08
MYOM2	Smooth muscle	−0.105851193	0.639193945
TCAP	Smooth muscle	−0.183046538	0.414859631

### 3.7 MELPHALAN and Testosterone Enanthate show stable binding affinity to MYOM2 and TCAP

**FIGURE 8:**
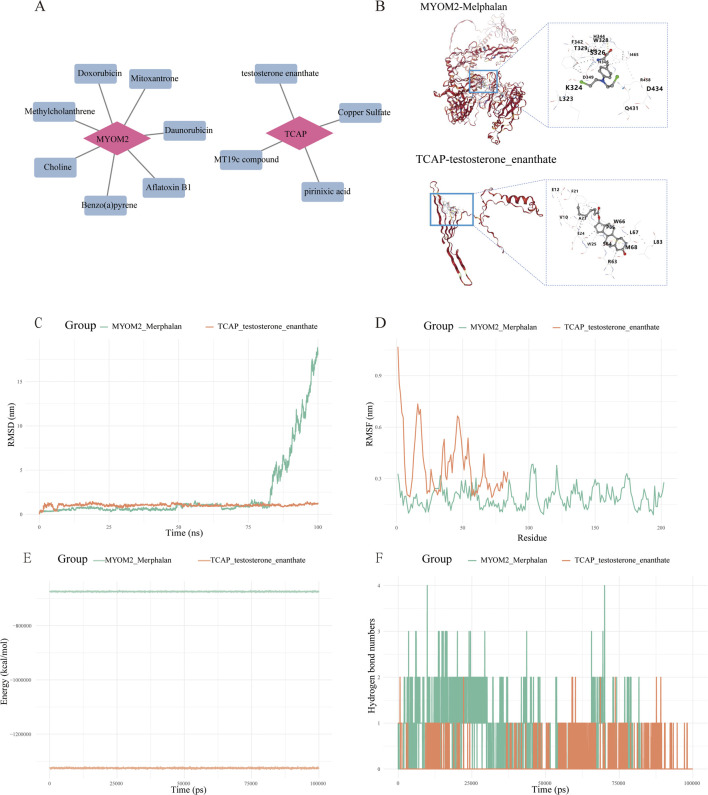
Hub genes exhibit favorable binding affinity with their corresponding compounds. **(A)** Key gene-compound interaction network, where hub genes are shown in purple and compounds are represented in blue modules. **(B)** The three-dimensional structure of the molecular ligand (compound) is displayed on the right; the left panel shows the visualization of the binding between the protein (key gene) and the molecular ligand (key active component), with different colors indicating distinct regions of the protein. **(C)** RMSD values of the complex during 0–100 ns simulation. **(D)** RMSF values of the complex in the 0-200 amino acid residue region. **(E)** Total energy of the complex, with the horizontal axis representing time and the vertical axis representing free energy. **(F)** Changes in the number of hydrogen bonds in the complex over the simulation time.

DSigDB is a specialized database dedicated to associating drugs/compounds with target genes and can be applied to GSEA. It contains a total of 22,527 gene sets, covering 17,389 unique compounds and 19,531 genes. Constructed based on data of quantitative drug-induced inhibition and gene expression alterations, the database is classified into four datasets. In the present study, this database was utilized to carry out relevant analyses including drug-gene association analysis and drug repositioning prediction. Analysis via the DSigDB (v. 1.0) predicted 10 and 3 compounds that interact with MYOM2 and TCAP, respectively, with no shared compounds identified between the two. Among these, MELPHALAN (for MYOM2) and Testosterone Enanthate (for TCAP) exhibited the highest combined scores and were thus selected as ligands for subsequent analyses (Figure 8A). Molecular docking results showed favorable binding capacities of these ligands to their respective targets, with binding free energies of −6.0 kcal/mol and −5.9 kcal/mol, respectively (Figure 8B). To validate the stability of the docked conformations, 100 ns molecular dynamics simulations were performed. RMSD analysis revealed that the TCAP-Testosterone Enanthate complex displayed minimal structural fluctuations throughout the simulation (RMSD maintained within 0–2.5 nm), while the MYOM2-MELPHALAN complex also exhibited similar stability during the 0–80 ns period (Figure 8C). RMSF analysis indicated that the MYOM2-MELPHALAN complex possessed higher flexibility in the 100–125 and 160–180 residue segments. In contrast, the TCAP-Testosterone Enanthate complex showed significant fluctuations in the 0–60 residue region, suggesting this segment may serve as the ligand-binding interface (Figure 8D). Both complexes exhibited low total energy with gentle fluctuations, indicating stable energy states of the systems (Figure 8E). The number of hydrogen bonds showed periodic changes during the simulation, with the TCAP-Testosterone Enanthate and MYOM2-MELPHALAN complexes maintaining 0–2 and 0-4 hydrogen bonds, respectively. This reflects a dynamic equilibrium at the binding interface and good elasticity of the hydrogen bond network (Figure 8F). Collectively, the results of molecular docking and dynamics simulations support that both the MYOM2-MELPHALAN and TCAP-Testosterone Enanthate complexes possess high structural stability.

### 3.8 RT-qPCR shows upregulation of MYOM2 and TCAP in OA

**FIGURE 9:**
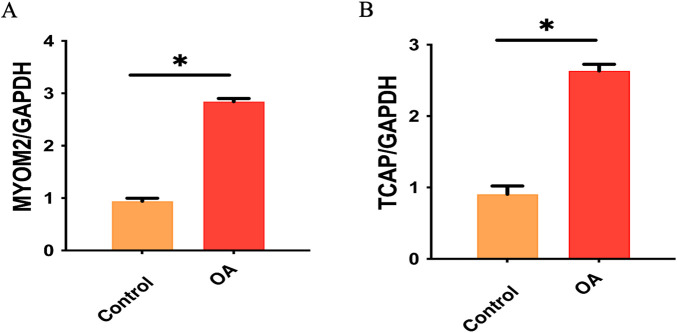
Validation of target gene expression by RT-qPCR. **(A)** MYOM2 expression in the control groups and the OA groups. MYOM2 expression was markedly elevated in the OA groups compared with the control groups. **(B)** TCAP expression in the control group and OA groups. TCAP expression was significantly upregulated in the OA group compared with the control group.

RT-qPCR was performed to compare the mRNA expression levels of MYOM2 and TCAP between the control group and osteoarthritis model group. The results showed that there were significant differences in the expression of these two genes (p < 0.05). Specifically, the expression level of MYOM2 was significantly increased in the osteoarthritis model group compared with the control group (p < 0.05) (Figure 9A). Similarly, the expression of TCAP was also remarkably upregulated in the model group relative to the control group (p < 0.05) (Figure 9B). Collectively, these findings further verified the authenticity and reliability of the bioinformatics analysis results. In addition, it also suggests that MYOM2 and TCAP, the key target genes of arginine and proline metabolism, are closely associated with the pathogenesis of OA.

## 4 Discussion

OA is a chronic degenerative joint disease with joint pain, swelling, and even joint mobility disorders. Its course is long, the disease is prolonged and difficult to cure, and the pathogenesis is not clear. Recent studies have shown that dysregulated arginine and proline metabolism within the joint is closely associated with OA pathogenesis. Metabolomic profiling of human OA synovial fluid has revealed significantly upregulated arginine and proline metabolism compared with healthy controls (Carlson et al., 2018). In OA cartilage from both human patients and mouse models, the arginine-metabolizing enzyme arginase II is specifically upregulated in chondrocytes; overexpression of arginase II in mouse joint tissues causes OA pathogenesis, whereas genetic ablation of arginase II abolishes surgically induced OA (Choi et al., 2019). Concurrently, multi-omics analysis of paired OA synovial tissue and fluid has detected enrichment of proline-related metabolic pathways and elevated hydroxyproline levels, reflecting active collagen degradation (Ge et al., 2025). These findings indicate that aberrant arginine and proline metabolism promotes synovial inflammation and cartilage destruction in OA. However, the specific genes through which this metabolic dysregulation drives OA pathogenesis have not been fully characterized. By using transcriptomics sequencing data and integrating with comprehensive gene expression databases for bioinformatics analysis, it has been widely applied to discover new therapeutic targets for diseases. Therefore, the focus of our research was to determine the diagnostic markers and potential therapeutic targets related to arginine and proline metabolism in OA. Through a systematic analysis, we unexpectedly discovered that MYOM2 and TCAP are key biomarkers and therapeutic targets related to arginine and proline metabolism in OA, ultimately influencing the progression of OA.

In this study, clinical synovial tissues from OA patients and normal control groups were collected for sequencing, and the differential analysis of sequencing data training sets of 12 OA patient samples and 10 normal control samples was performed. A total of 2,227 DEGs were screened out, including 1,435 upregulated genes and 792 downregulated genes, which indicated that OA was involved in multiple complex biological processes (Chen and Zhang, 2024). In the GO and KEGG enrichment analysis, 96 candidate genes of arginine and proline metabolism were found to be related to a series of biological processes and signaling pathways of muscle tissues and functions, especially key functions such as muscle organ development, muscle system process, and muscle tissue development. This indicates that muscle-specific kinase (MUSK)-related genes and their functions may play an important role in maintaining the motor function and structural stability of joints during OA progression, and also suggests that abnormal muscle function may be an important factor affecting the function and structural stability of joints during OA progression (Shorter et al., 2019; Krishnasamy et al., 2018). These results not only deepen our understanding of OA pathogenesis, but also suggest that new therapeutic strategies can be developed in the future by modulating muscle and related signaling pathways.

This study found that MYOM2 and TCAP are the key biomarkers of arginine and proline metabolism in OA, which play an important role in the occurrence and development of OA. The TCAP gene plays a key role in maintaining muscle structure and function. The TCAP protein encoded by the gene interacts with the giant Titin to regulate the elasticity and stability of muscle fibers (Zhao et al., 2024). TCAP not only participates in the regulation of muscle contraction, but also plays an important role in cytoskeleton signaling and mechanical force sensing (Lv et al., 2023; Knöll et al., 2011). For patients with OA, the injury and degeneration of muscle tissue around the joint is a common phenomenon, and the loss of muscle function may further aggravate the wear and inflammation of the joint (Yang et al., 2023). Abnormal expression or dysfunction of TCAP gene may accelerate the development of OA by affecting the structural integrity and ability of muscle contraction, thereby leading to decreased joint stability (Hodge et al., 2019). Therefore, TCAP gene plays an important role in the maintenance of joint and muscle function, and its regulatory role in OA deserves further study in order to provide new molecular targets for the treatment of OA. MYOM2 is essential for maintaining the structural integrity of muscle cells and responding to mechanical stress (Woods et al., 2008). The MYOM2 encoded by this gene is a muscle-specific structural protein located at the M-line of muscle fibers and mainly involved in regulating the mechanical stability and elasticity of muscles (Wang et al., 2024). MYOM2 is critical in maintaining the structural integrity of muscle cells and responding to mechanical stress. In OA, the muscle tissue around the joint is often damaged or degraded, and the weakening of muscle function may further aggravate the degenerative changes and inflammatory response of the joint. The abnormal expression or dysfunction of MYOM2 gene may affect the structural stability of muscle fibers, leading to the dysfunction of muscles around the joint. Thereby it reduces the ability of the joint to support and accelerates cartilage damage and joint degradation (Liu et al., 2019). The MYOM2 gene plays an important role in regulating muscle and joint stability, and its potential regulatory mechanisms may provide new molecular targets for OA intervention.

The shared enrichment of TCAP and MYOM2 in neurodegenerative disease pathways suggests that these hub genes may participate in neurobiological processes extending beyond the joint. Emerging evidence indicates that OA involves not only local joint degeneration but also peripheral and central nervous system sensitization. Within OA joints, nociceptive afferent neurons are sensitized by neuropeptides, neurotrophins, and pro-inflammatory cytokines released from inflamed synovium and degrading cartilage, leading to chronic pain signaling (Batchelor et al., 2025). Beyond peripheral sensitization, a growing body of literature has established epidemiological and mechanistic links between OA and central neuroinflammation. A recent review demonstrated that OA-associated systemic inflammation can compromise blood-brain barrier integrity and exacerbate neuroinflammatory processes, while shared mechanisms such as oxidative stress and mitochondrial dysfunction connect OA with neurodegenerative conditions including Alzheimer’s and Parkinson’s diseases (Rabie et al., 2025). The co-enrichment of MYOM2 and TCAP in Alzheimer’s, Parkinson’s, and Huntington’s disease pathways identified in our GSEA analysis may therefore reflect these shared cellular stress and metabolic dysregulation mechanisms at the systems level. Importantly, central sensitization and neuropathic-like pain have been recognized as key drivers of chronic OA pain, with studies showing that central sensitization-related signs are present in a substantial proportion of OA patients and are independent predictors of poor functional outcomes (Salaffi et al., 2025). This has therapeutic implications, as interventions targeting central pain mechanisms rather than peripheral nociception alone are increasingly recognized as necessary components of comprehensive OA management. Furthermore, the enrichment of TCAP and MYOM2 in cardiac muscle contraction pathways, together with their predominant expression in skeletal muscle and heart (GTEx Consortium., 2020), raises the possibility that these genes participate in a broader musculoskeletal-cardiovascular regulatory network that may be coordinately dysregulated in OA, a disease known to share risk factors and comorbidities with cardiovascular disorders. Further investigation into whether MYOM2 and TCAP modulation affects neural sensitization pathways or muscle-joint-neural crosstalk may reveal novel therapeutic strategies targeting both structural joint pathology and chronic pain in OA. Analysis of immune cell infiltration also revealed that TCAP and MYOM2 showed significant correlations with endothelial cells and skeletal muscle cells, which was consistent with the GSEA enrichment results. These findings further support the potential involvement of these two genes in skeletal muscle and cardiovascular functions during OA pathogenesis, thereby providing new directions for mechanistic investigations of this disease.

This study collected clinical samples from OA patients and normal controls, and performed quantitative RT-qPCR analysis on key targets. The results showed that MYOM2 and TCAP were significantly upregulated in OA synovial tissues, further suggesting the important roles of the screened MYOM2 and TCAP in OA progression. While these findings reinforce the potential of MYOM2 and TCAP as biomarkers for OA, their specificity requires further validation. Although our GSEA analysis revealed that these genes are co-enriched with Alzheimer’s and Huntington’s disease pathways, GTEx data demonstrate that both MYOM2 and TCAP are predominantly expressed in skeletal muscle and heart, with minimal expression detected in the central nervous system (GTEx Consortium., 2020). This pathway-level co-enrichment more likely reflects shared cellular stress and metabolic dysfunction mechanisms rather than genuine expression of these genes in neural tissues. Another consideration is whether MYOM2 and TCAP are similarly dysregulated in other inflammatory joint diseases, as this would directly affect their diagnostic specificity. Currently, publicly available datasets do not provide direct comparisons of MYOM2 or TCAP expression in synovial tissues from patients with other acute inflammatory arthropathies. However, expression data from non-synovial sources offer informative contrasts. RNA sequencing of peripheral blood mononuclear cells from patients with ankylosing spondylitis revealed that MYOM2 is significantly downregulated compared with healthy controls (Huang et al., 2020), a direction of change opposite to the upregulation observed in OA synovial tissue in the present study. This divergent expression pattern suggests that MYOM2 dysregulation is disease-context-dependent rather than a universal feature of inflammatory arthritis. Furthermore, a single nucleotide polymorphism of MYOM2 has been reported to be significantly associated with responsiveness to etanercept treatment in ankylosing spondylitis patients (Liu et al., 2019). Collectively, the existing evidence supports the potential of MYOM2 and TCAP as relatively specific indicators of OA-related joint pathology. Systematic profiling of these genes across synovial tissues from multiple inflammatory joint diseases remains an important direction for future validation.

The study also predicted potential therapeutic agents targeting two hub genes. The analysis revealed that MYOM2 may potentially interact with 10 small-molecule drugs, among which MELPHALAN achieved the highest comprehensive score for MYOM2. Molecular docking and molecular dynamics simulations showed its strong target binding affinity and structural stability. Clinically, MELPHALAN is a nitrogen mustard alkylating agent used in the treatment of multiple myeloma and ovarian cancer. Its antitumor activity is mediated through DNA interstrand cross-linking at the N7 position of guanine, leading to inhibition of DNA replication and induction of apoptosis in rapidly dividing cells (Esma et al., 2017). Notably, the biological effects of MELPHALAN are dose-dependent: at concentrations approximately 100-fold lower than cytostatic doses, MELPHALAN loses its DNA-damaging activity and instead disrupts signal transduction through TNF-α and IL-2β receptors, thereby exerting anti-inflammatory effects independent of its alkylating properties, as evidenced by reduced severity of experimental colitis in mice following non-cytotoxic dosing (Pukhalsky et al., 2006); conversely, at cytotoxic doses MELPHALAN induces oxidative stress, inflammation, and apoptosis through modulation of pro-inflammatory cytokines and apoptotic markers (Panghal et al., 2023). These findings collectively establish that MELPHALAN can exert dose-dependent regulation of cell proliferation, apoptosis, and inflammatory signaling, suggesting that MYOM2 in non-oncological conditions such as OA may be involved in analogous regulatory pathways. The analysis also indicated that TCAP is associated with three drugs, primarily involved in hormonal regulation, anti-inflammatory effects, and metabolic pathways. Among them, Testosterone Enanthate scored the highest for TCAP. As a hormonal regulator, it may potentially modulate muscle function and promote tissue repair, which holds significant implications for the clinical treatment of OA (Howard et al., 2022). The construction of a drug-gene interaction network revealed that regulating pathways associated with MYOM2 and TCAP could lead to the development of novel therapeutic strategies for OA. The association between MYOM2 and chemotherapeutic agents suggests that inhibiting specific cellular metabolic and proliferative pathways may influence the pathological progression of OA. Meanwhile, the link between TCAP and anti-inflammatory or hormone-regulating drugs implies that modulating joint inflammation and tissue repair through these pathways could offer more targeted treatment options for patients. We employed computational drug prediction and molecular binding validation to assess the pharmacological relevance of these targets. These findings provide compelling new leads for the development of more effective OA therapies, with the potential to reduce drug development costs and advance personalized medicine. This study contributes to the field by highlighting the significance of these promising therapeutic targets in OA treatment.

Although our study provided valuable insights, several limitations should be acknowledged. Our basic experiments focused on validation at the gene expression level and did not include functional assays. Furthermore, the molecular docking and dynamics simulations conducted in this study rely heavily on the quality of the three-dimensional structures of the proteins and ligands. Although such computational analyses can offer important clues for drug target identification, they are insufficient to fully predict the actual effects of these targets in complex physiological environments. Subsequent validation—from *in vitro* and *in vivo* studies to eventual clinical trials—is essential to confirm the therapeutic potential of these targets. Such efforts will help to better understand the roles of arginine and proline metabolism in OA and facilitate the clinical translation of related research findings.

In conclusion, this study collected clinical samples for transcriptome sequencing and combined it with multiple bioinformatics techniques to identify TCAP and MYOM2 as hub genes involved in arginine and proline metabolism in OA. These genes are key biomarkers and potential therapeutic targets, and these targets were further validated through *in vitro* experiments. Furthermore, pharmacological prediction, molecular docking, and molecular dynamics simulations not only verified the feasibility of these key targets at the pharmacological level but also screened promising lead compounds. These findings provide candidate drugs and a theoretical framework for interventional therapy of OA, holding significant potential for accelerating the development of cost-effective and highly efficient personalized treatment strategies. This study may offer new directions for precision medicine in OA.

## Data Availability

The datasets presented in this study can be found in online repositories. The names of the repository/repositories and accession number(s) can be found in the article/Supplementary Material.
