# Supplementary material for: Identification of potential biomarkers and therapeutic targets for osteoarthritis associated with arginine and proline metabolism based on transcriptome sequencing and bioinformatics
Source: Front Genet. 2026 Jul 3;17:1795267. doi: 10.3389/fgene.2026.1795267 (PMC13375187; doi:10.3389/fgene.2026.1795267)
Supplement: Supplementary file 1 [file DataSheet1.PDF]

Supplementary Material

1 Supplementary Figures

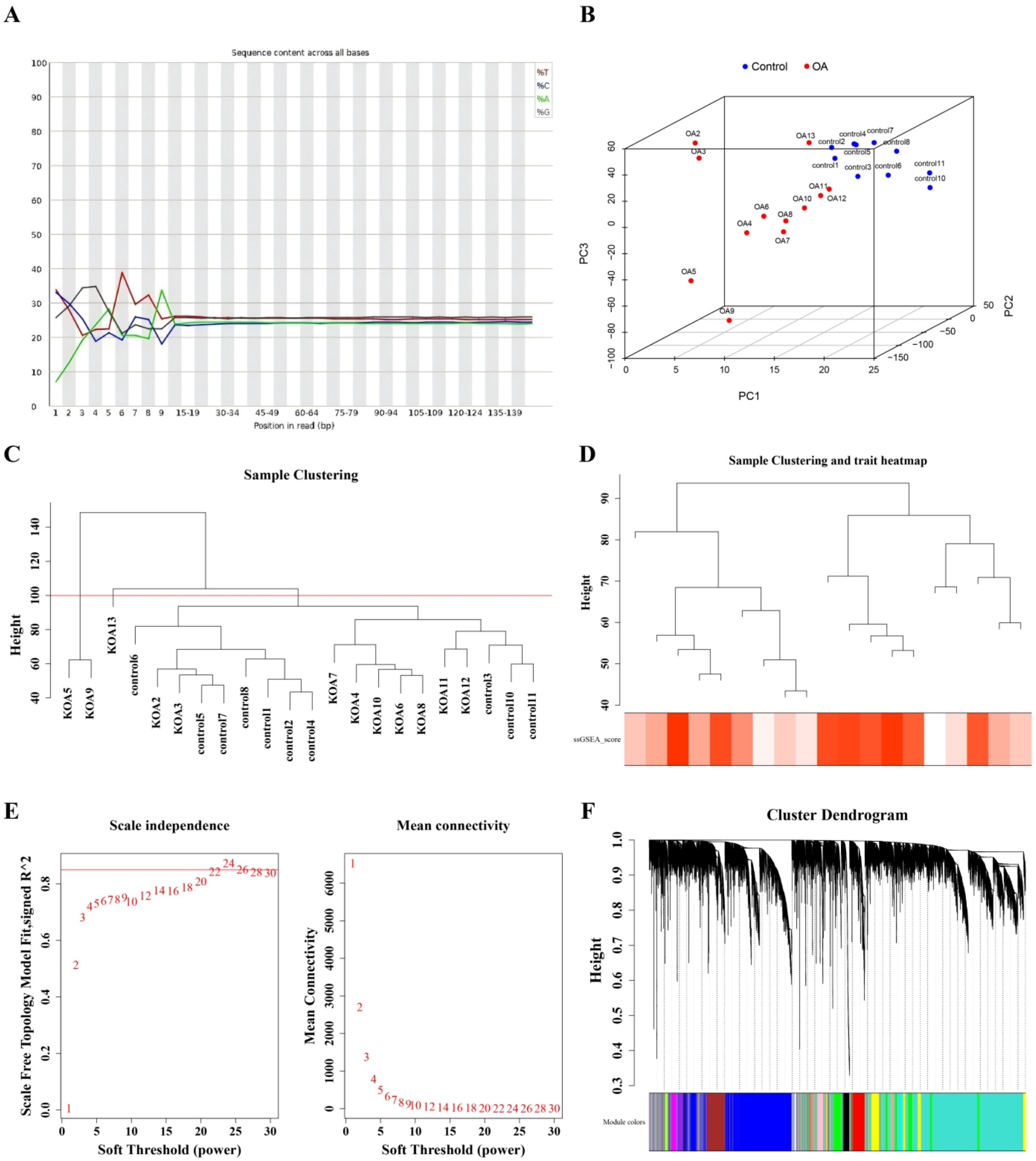

Supplementary Figure 1. Identification of key module genes related to arginine and proline metabolism in OA. (S1A) Distribution of ATCG content. (S1B) PCA analysis of control and OA samples. It showed evident separation of samples between OA and control groups. (S1C-D)

Hierarchical clustering diagram. After removing outliers, APRG ssGSEA scores were recalculated for each sample, followed by re-performing clustering analysis. (S1E) Scatter plot of power values. A soft threshold of $R^2 = 0.85$ was set. A power of 24 generated a scale-free network with low mean connectivity, which was defined as the optimal soft threshold β. (S1F) Cluster tree diagram combined with similar modules.It identified 17 modules correlated with the ssGSEA scores of APRGs.

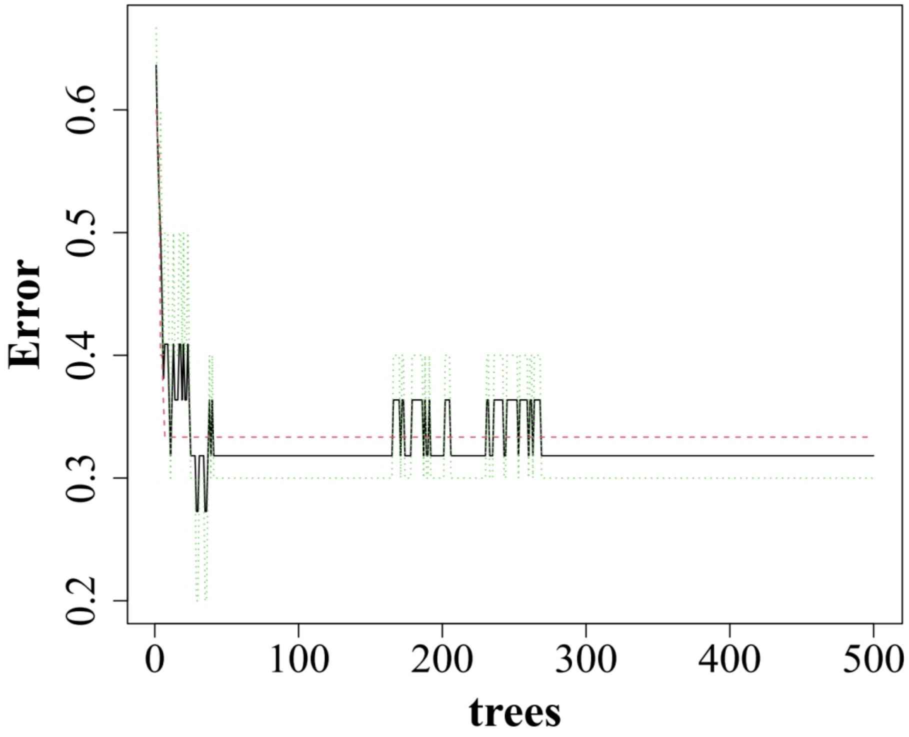

Supplementary Figure S2. Variable importance analysis from random forest model.Variable importance plot of all 23 candidate genes derived from the random forest model, ranked by mean decrease in Gini index, reflecting their relative importance in classification performance.
